# Rising from the Ashes: DNA Repair in *Deinococcus radiodurans*

**DOI:** 10.1371/journal.pgen.1000815

**Published:** 2010-01-15

**Authors:** Michael M. Cox, James L. Keck, John R. Battista

**Affiliations:** 1 Department of Biochemistry, University of Wisconsin-Madison, Madison, Wisconsin, United States of America; 2 Department of Biomolecular Chemistry, University of Wisconsin-Madison, Madison, Wisconsin, United States of America; 3 Department of Biological Sciences, Louisiana State University and A & M College, Baton Rouge, Louisiana, United States of America; Baylor College of Medicine, United States of America

The extraordinary resistance of *Deinococcus radiodurans* to ionizing radiation (IR) and desiccation is slowly drawing more intense scrutiny. Relative to most other organisms, *Deinococcus* has a survival advantage measured in orders of magnitude. Exposure to 5 kGy of IR reduces the genome of any bacterium to hundreds of fragments. *Deinococcus* is no exception. However, *Deinococcus* seems to take this catastrophe in stride. Over a period of 3–4 hours, overlapping fragments are spliced together into complete chromosomes, and the cells soon resume normal growth. There is no measurable lethality. Attempts to understand the molecular basis of this phoenix-like capability has given rise to numerous hypotheses. Notable among them are proposals that the condensed nature of the *Deinococcus* genome [1] or an unusual capacity to avoid protein oxidation [2] are keys to radiation resistance.

Regardless of the physiological or metabolic adaptations that *Deinococcus* may employ to enhance survival, it is hard to explain extreme genome reconstitution without considering DNA repair. As originally defined by Daly and Minton [3], and reinforced more recently by Radman and colleagues [4],[5], genome reconstitution in *Deinococcus* proceeds in two phases. The first phase has been attributed to a process dubbed extended synthesis-dependent single-strand DNA annealing (ESDSA) [4],[5]. The second phase involves RecA protein-mediated double-strand break repair. Some initial studies suggested that the first phase of repair did not involve the *Deinococcus* RecA protein, but more recent work has documented a role for RecA in both phases [4],[5]. ESDSA involves considerable nuclease activity to generate single-stranded DNA, strand invasion mediated by the RecA and/or RadA proteins, and extensive DNA synthesis primed by the invading strands prior to the annealing steps [4],[5].

**Figure 1:**
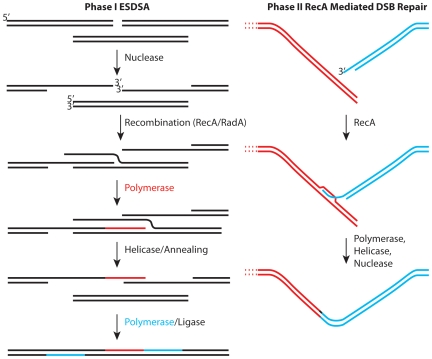
Two stages of genome reconstitution in *Deinococcus radiodurans*. The first stage, extended synthesis-dependent single-strand annealing (ESDSA) is dominated by nuclease and DNA polymerase functions. The second stage is a more conventional RecA-mediated double-strand break repair process focused on the final splicing of large chromosomal segments.

The emerging picture (Figure 1) provides a useful framework, but one with many questions. Most of these involve enzymes and their roles. The generation of single-stranded DNA in ESDSA requires the function of at least one nuclease and helicase and perhaps several of both. Proteins are also needed to load RecA protein onto single-strand DNA binding protein (SSB)-coated single-stranded DNA. The most important pathway for double-strand break repair in *Escherichia coli* utilizes the RecBCD enzyme for all of these roles. However, *Deinococcus* encodes no homologue of RecB or RecC. *Deinococcus* does encode homologues of every protein involved in what is considered (in *E. coli*) an auxiliary pathway for recombinational DNA repair—the RecFOR pathway. In the RecFOR pathway, the RecJ and RecQ proteins take up the nuclease and helicase roles, respectively, while the RecFOR proteins function to load RecA protein onto the DNA. The absence of RecBC homologues in *Deinococcus* seems to imply that the RecFOR path is particularly important.

In two important reports in this issue of *PLoS Genetics*, Sommer and colleagues convert much recent speculation into substance. In the first report [6], the Chandler and Sommer laboratories collaborate to explore the mechanism of transposition of element IS*Dra2*. This transposon is a member of a family of elements that transpose via single-stranded DNA intermediates. Transposition is activated by irradiation of *Deinococcus*. The work not only documents the transposition mechanism, it reinforces the proposition that extensive lengths of single-stranded genomic DNA are generated in the early stages of genome reconstitution in this bacterium. As a bonus, the work provides hope for the development of in vivo transposition as a tool for genetic manipulation of this genome.

The second report [7] clearly establishes the central role of the RecFOR pathway in genome reconstitution. Where the effects of RecFOR pathway gene deficiencies are generally modest in *E. coli*, they are dramatic in *D. radiodurans*. Sommer and colleagues demonstrate that cells lacking functional *recF*, *recO*, or *recR* genes are essentially as dysfunctional in genome reconstitution as *recA* mutants. Cells with these deficiencies are viable in the absence of extreme DNA damage, but grow much slower than wild type. Thus, the RecFOR pathway is important during normal replication, as well as during genome reconstitution. If the gene encoding the RecJ nuclease is inactivated, the cells are inviable. Surprisingly, cells lacking the RecQ helicase exhibit wild type resistance to IR. Instead, the helicase that appears to be critical is UvrD. In *E. coli*, the function of UvrD in recombinational DNA repair is to remove RecA filaments from the DNA [8],[9]. In *Deinococcus*, it almost certainly does more. In summary, *Deinococcus* now presents a unique opportunity to demonstrate what the RecFOR pathway can really do.

This RecFOR pathway may look a bit different from its cousin in *E. coli*. After irradiation, about 60 *Deinococcus* genes are induced, and quite a number of them have roles in genome reconstitution [10]. Many of these genes are novel. Knockouts do not have the dramatic effects of *recAJFOR* knockouts, but their cumulative effects can be significant. Some, like the DdrA protein (a distant homologue of the eukaryotic Rad52 protein [11]), and DdrB (a novel single-strand DNA binding protein [12]) must be worked into the schemes.

The work of Chandler and Sommer has a few more far-reaching implications. The spectacular feat of genome reconstitution after heavy irradiation does not require a completely new pathway for double-strand break repair, and no such pathway appears to be present. Instead, *D. radiodurans* relies heavily on a set of recombinational DNA repair functions that are recognizable in almost all species. In large measure, efficient genome reconstitution involves tweaking those repair proteins, providing a few novel augmentations, and perhaps modifying the environment in which all of these proteins function. However, the properties already noted that distinguish the RecFOR pathway in *Deinococcus* from the same process in *E. coli* bear reiteration. The roles that well-known repair proteins play in radioresistance are not perfectly predictable, based on what we understand about their function in *E. coli*. An orthologous relationship between proteins can inform speculation, but it must be subjected to experimental substantiation. Every DNA repair protein examined to date in *D. radiodurans* has provided one or more novel twists in our understanding of its function, structure, interaction with other proteins, and role in repair.

Last but not least, we may soon see a first in vitro reconstitution of a complete DNA double-strand break repair reaction. In this arena, *D. radiodurans* is gradually eclipsing *E. coli* as the most pliable bacterial model system. The proteins, or at least most of them, are in hand. Fortuitously, the enzymes from *Deinococcus* appear to be more amenable to structural determination than their *E. coli* cousins. The only structural information currently available about RecF, RecO, and RecR come from the *Deinococcus* enzymes [13]–[17]. Activities are being characterized, and more surprises are anticipated. *Deinococcus* simply does it better.
